# Application of a low‐cost Geiger–Müller monitoring system for clinical radiation environments

**DOI:** 10.1002/acm2.70744

**Published:** 2026-08-13

**Authors:** Jungsam Choi, Chai Hong Rim, Hakyoung Kim, Wonho Lee

**Affiliations:** ^1^ Department of Radiation Oncology Korea University Guro Hospital, Korea University College of Medicine Seoul Republic of Korea; ^2^ Department of Radiation Oncology Korea University Ansan Hospital, Korea University College of Medicine Seoul Republic of Korea; ^3^ School of Health and Environmental Science Korea University Seoul Republic of Korea

**Keywords:** calibration coefficient, Geiger–Müller detector, radiation monitoring, radiation protection, survey meter

## Abstract

**Background:**

Low‐cost Geiger–Müller detectors may support clinical radiation monitoring, but their response varies with radiation type and measurement conditions.

**Purpose:**

To evaluate an Arduino‐based Geiger–Müller monitoring system in representative clinical radiation environments and determine whether condition‐specific calibration reduces disagreement with a reference survey meter.

**Methods:**

The system was compared with a reference energy‐compensated Geiger–Müller survey meter (Ludlum Model 3005) under computed tomography scatter, Ir‐192 brachytherapy, and 6 MV linear accelerator maze conditions. Calibration coefficients, expressed as counts per minute (CPM) per µSv/h, were estimated using zero‐intercept regression, and method agreement was assessed using bias analysis and Bland–Altman plots. Portable deployment and user acceptability were also evaluated. The effective dead time of the counting chain was estimated using the first‐order two‐source approximation, and the intrinsic photon energy response was characterized using discrete‐energy gamma‐emitting sources (Co‐57, Cs‐137, and Co‐60). Calibration‐coefficient stability was also assessed at a fixed maze position across nominal dose rates of 100–600 MU/min to isolate count‐rate effects.

**Results:**

Calibration coefficients ranged from 124 to 133 for computed tomography scatter, 99 to 179 for Ir‐192, and 145 to 233 for the 6 MV linear accelerator maze, demonstrating marked condition dependence. Applying condition‐specific calibration reduced systematic bias within the evaluated environments. The effective dead time was approximately 258 µs, and the detector showed greater response per unit dose at lower photon energies, consistent with the known energy dependence of uncompensated Geiger–Müller tubes and providing a plausible explanation for the condition‐dependent calibration coefficients. At a fixed maze position, the calibration coefficient varied little across nominal dose rates of 100–600 MU/min (coefficient of variation, 3.04%), with no evidence that count‐rate dependence dominated within this range. In the linear accelerator maze, mean bias approached zero after distance‐specific calibration, whereas the limits of agreement widened with increasing distance. Among 27 respondents, intention for continued use was more strongly associated with perceived stability and lower perceived risk than with numerical agreement alone.

**Conclusions:**

With condition‐specific calibration, the low‐cost monitoring system showed low mean bias relative to the reference instrument in the evaluated low‐to‐moderate dose‐rate clinical environments. These findings support further evaluation of the system as a supplementary tool for routine radiation monitoring and safety checks under comparable clinical conditions.

## 1 INTRODUCTION

Medical radiation is integral to diagnostic imaging and radiation therapy. Accurate radiation measurement is fundamental to quality assurance (QA), patient safety, and occupational protection.^1^, ^2^, ^3^, ^4^ Clinical radiation measurements use various instruments, including ionization chambers, scintillation detectors, semiconductor detectors, personal dosimeters, and survey meters.^5^, ^6^, ^7^ Survey meters are widely used for leakage monitoring and routine safety checks because they are portable, practical, and suitable for periodic calibration.^3^, ^5^, ^8^

Despite these advantages, commercial survey meters may be difficult to deploy extensively in research, education, and resource‐constrained settings. Their cost, limited scalability for distributed deployment, and restricted automated data‐logging capabilities can hinder broader implementation, particularly when repeated measurements are required at multiple clinical locations during the same session.^4^, ^9^, ^10^, ^11^ These constraints are particularly relevant because radiation‐safety workflows often require repeated measurements under varied clinical conditions.^3^, ^9^

Low‐cost, open‐source radiation measurement systems combining Arduino‐based platforms with Geiger–Müller (GM) tubes have therefore attracted increasing interest.^11^, ^12^, ^13^, ^14^, ^15^ Arduino provides an accessible and scalable hardware–software platform, whereas GM tubes permit relatively simple pulse‐based radiation detection.^6^, ^13^, ^15^ Such systems may offer practical supplementary tools for continuous multipoint area monitoring, which can be cost‐prohibitive with standard instrumentation, provided their performance is rigorously evaluated in realistic medical radiation environments.^11^, ^14^, ^15^ However, uncompensated GM tubes inherently exhibit marked energy dependence. This dependence can cause substantial measurement error across the diverse energy spectra encountered clinically, from low‐energy x‐ray scatter in computed tomography (CT) to high‐energy secondary radiation in linear accelerator (LINAC) mazes. Accordingly, evaluations against calibrated reference meters in clinical environments remain limited. Condition‐specific calibration coefficients (CFs), expressed as counts per minute (CPM) per µSv/h, remain insufficiently characterized across variations in energy spectrum, measurement geometry, and shielding conditions.^5^, ^6^, ^15^

Accordingly, we evaluated an Arduino‐based Geiger–Müller (ABG) monitoring system through simultaneous measurements with a reference energy‐compensated GM survey meter (Ludlum Model 3005). Measurements were obtained under representative clinical radiation conditions, including CT scatter, Ir‐192 brachytherapy, and a 6 MV LINAC maze. Using paired measurements, condition‐specific CFs were derived and evaluated for converting count‐based outputs into dose‐rate units. We also examined count‐rate‐dependent under‐response at higher count rates, estimated effective dead time using the first‐order two‐source method, and characterized intrinsic photon energy response using discrete‐energy gamma sources to investigate mechanisms underlying the observed condition dependence. In addition, we assessed the practical utility of a portable system through a survey of hospital staff working in medical radiation environments.

This study aimed primarily to derive condition‐specific CFs for the ABG system and assess its agreement with a reference energy‐compensated survey meter. Secondary objectives were to characterize the intrinsic energy response and effective dead time of the counting chain, examine performance limitations across dose‐rate conditions, and assess practical feasibility in representative clinical radiation environments.

## 2 MATERIALS AND METHODS

### 2.1 System configuration and reference instrument

**FIGURE 1:**
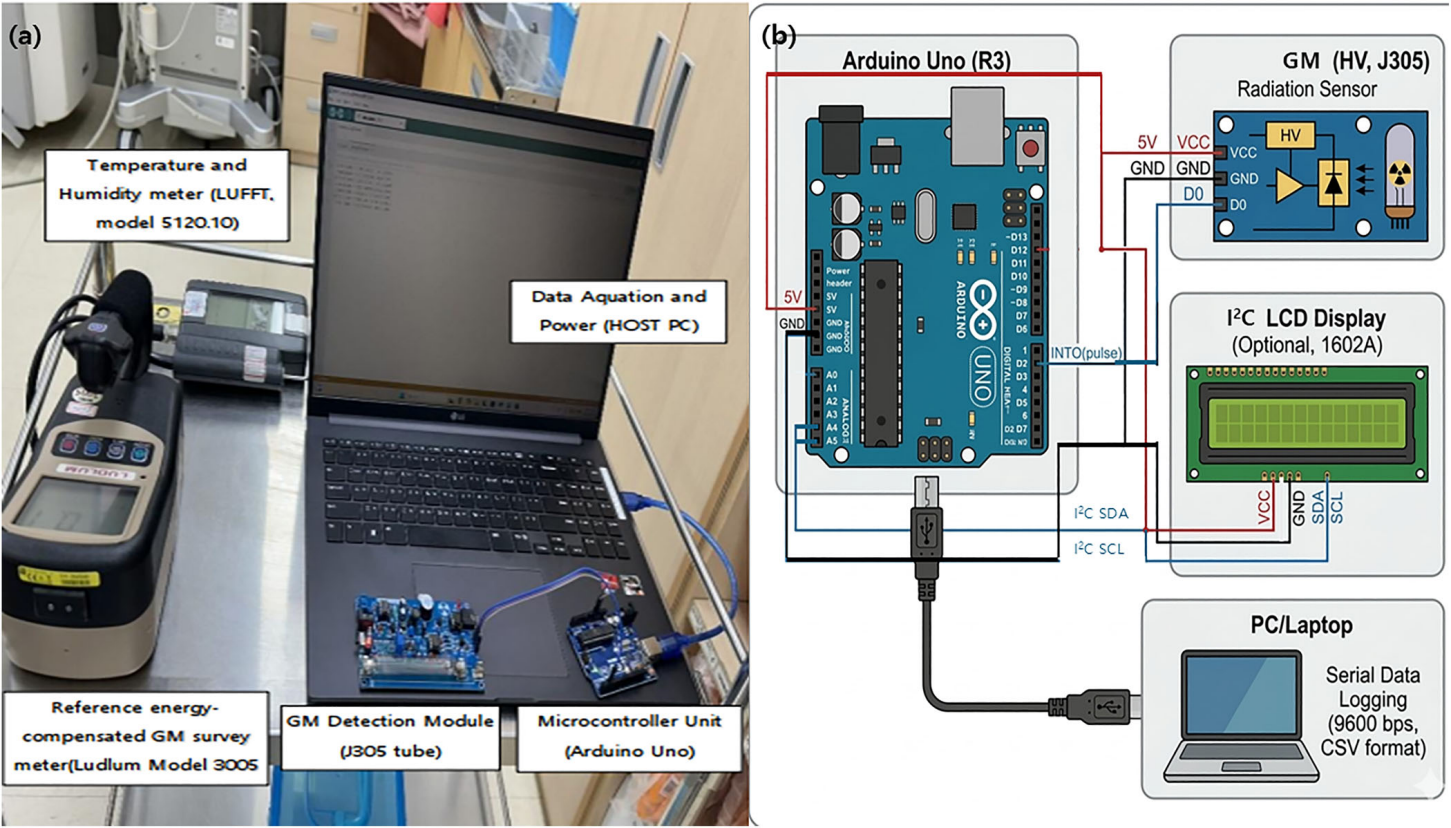
Experimental setup and architecture of the ABG monitoring system. (a) Simultaneous measurement setup showing the reference GM survey meter and ABG system positioned for concurrent measurements. (b) Hardware architecture of the ABG monitoring system.

The ABG monitoring system comprised an Arduino Uno R3 microcontroller (ATmega328P), a J305 GM tube module, an optional 1602A liquid crystal display (LCD), and a laptop‐based USB–serial logging system (Figure 1; Table 1).^6^, ^13^

**TABLE 1:** Core hardware components and operating settings of the ABG monitoring system.^d^

Component	Model/version	Key specifications	Operating settings in this study
GM tube module	J305 GM tube module^a^	Coaxial cylindrical GM tube; operating voltage, 380–450 V; gamma sensitivity, ≥0.1 MeV; intrinsic background, approximately 0.2 pulses/s; minimum plateau length, ≥80 V; maximum plateau slope, ≤10% per 80 V	Manufacturer‐default high‐voltage setting of approximately 420 V within the central plateau region; TTL output pulse transmitted to the Arduino Uno external interrupt pin (D2); 1‐s counting interval
Microcontroller	Arduino Uno R3^b^ (ATmega328P)	16‐MHz clock; 5‐V logic; USB–serial interface; external interrupt INT0 (D2) available for pulse counting	Firmware developed in the Arduino integrated development environment; external interrupt pin D2 used for GM pulses; serial output to the PC at 9,600 bps; 5‐V power supplied through USB
Display (optional)	1602A LCD (16 × 2, I^2^C)^c^	I^2^C interface module (PCF8574); 5‐V supply; adjustable backlight and contrast	Updated every 1 s in synchronization with the counting interval; displays dose rate (µSv/h), CPM, and the measurement label or location
Logging/power	PC/laptop with USB–serial connection	5‐V USB power; serial communication through a virtual COM port	Logs timestamped CPM and dose‐rate data in CSV format; system clock synchronized before each measurement session

Abbreviations: CSV, comma‐separated values; I^2^C, inter‐integrated circuit; IDE, integrated development environment; PC, personal computer. Key specifications were obtained from the manufacturers’ datasheets unless otherwise indicated.

^a^ Specifications were obtained from the manufacturer's datasheet for the J305 GM tube module.

^b^ Specifications were obtained from the official technical documentation for the Arduino Uno R3.

^c^ Specifications were obtained from the manufacturer's datasheet for the 1602A LCD module with an I^2^C interface.

^d^ Preliminary functionality of the assembled system was verified before clinical measurements (Section 2.1).

The system was powered through a laptop USB connection at 5 V; TTL‐level pulses from the GM module were counted through the Arduino external interrupt pin (D2) in 1‐s intervals, and CPM was calculated by multiplying the 1‐s count by 60. Before clinical measurements, preliminary checks confirmed stable source‐free background counts, reliable TTL‐pulse detection, uninterrupted serial logging, and reproducible count‐rate responses across repeated measurements, verifying hardware operation and data‐acquisition stability rather than characterizing the intrinsic detector response. Alternative counting and averaging intervals were not evaluated. Table 1 summarizes the principal hardware components and operating settings.

The reference instrument was an energy‐compensated GM survey meter (Model 3005; Ludlum Measurements Inc., USA).^8^, ^16^ The reference meter was calibrated by ORBITECH Co., Ltd. on October 21, 2025 (certificate no. 2025‐D‐5571). During on‐site measurements, the meter was operated in dose‐rate mode (µSv/h) using the auto‐ranging and Auto‐FAST response settings.^8^ At each location, the ABG system and reference survey meter were positioned side by side at the same height and with minimal separation to reduce spatial variation.

### 2.2 Experimental scenarios

All measurements were performed indoors, with both devices positioned approximately 1 m above floor level. Each measurement condition was repeated three times. For the calibration and agreement analyses, background‐corrected paired 1‐s observations from all repetitions were pooled, with nonpositive net values excluded as described in Section 2.5. The reported N represents the number of paired observations retained for analysis. Representative dose rates were summarized as the mean of the retained observations, whereas mean bias was calculated as the mean of the paired differences between the ABG‐estimated and reference dose rates.

CT scatter measurements were obtained using an Aquilion Lightning 80 scanner (Canon Medical Systems) operated at 120 kV and tube currents of 15, 20, 25, 30, 35, 40, and 45 mA. The ABG system and reference meter were positioned in the patient‐feet direction at distances of 1 and 2 m from the bore entrance, with both detectors aligned with the table height. A continuous 30‐s exposure was delivered with the couch stationary and the gantry not rotating.

Ir‐192 measurements were performed in a maze‐type high‐dose‐rate brachytherapy suite using a GammaMed Plus afterloader (Varian Medical Systems). The source activity at the time of measurement was 3.21 Ci. Two configurations were evaluated: a sealed‐source near‐field configuration inside the treatment room at distances of 25, 50, 100, and 150 cm from the source and a covered maze‐exit scatter configuration in the corridor outside the maze‐exit door at distances of 100 and 200 cm. In both configurations, the ABG system and reference meter were positioned side by side at the same height.

**FIGURE 2:**
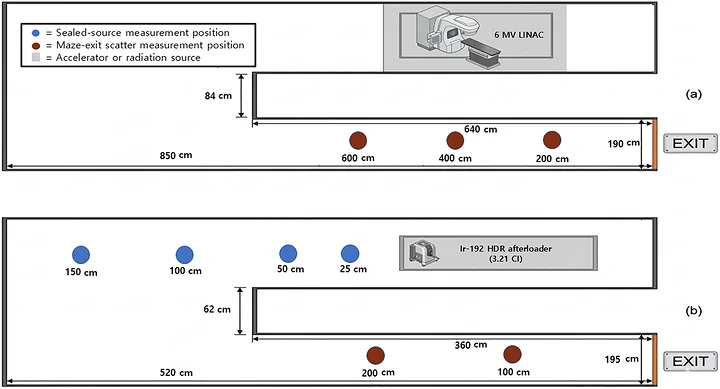
Measurement geometries used in the clinical evaluation. (a) Configuration of the 6 MV LINAC treatment room and maze. (b) Ir‐192 measurement setup using a sealed source with an activity of 3.21 Ci. Top view; not to scale.

The 6 MV LINAC maze measurements were performed using a Varian TrueBeam system with 6 MV photons, a 10 × 10 cm^2^ field, and a nominal dose rate of 400 MU/min. The nominal dose rate of 400 MU/min was selected because it is commonly used clinically and permitted repeated measurements under controlled, standardized irradiation conditions. The 10 × 10 cm^2^ field served as a standardized baseline for evaluating the reproducibility and condition‐specific response of the ABG system under controlled irradiation conditions. This setup was designed to characterize the ABG system under a representative clinical irradiation condition rather than under a maximum‐scatter shielding‐survey configuration. The gantry angle was 0°, and the couch was positioned at the isocenter without a phantom in the beam. Maze measurements were obtained at distances of 2, 4, and 6 m from the maze‐exit door, with both devices positioned side by side 1 m above floor level. To further examine the influence of count‐rate dependence on the CF, an additional dose‐rate analysis was performed at the fixed 2‐m LINAC maze position. The ABG system and calibrated reference survey meter remained side by side at a fixed location while measurements were obtained using 6 MV photons and a 10 × 10 cm^2^ field. The nominal dose rate was varied from 100 to 600 MU/min in increments of 100 MU/min, yielding six settings that bracketed the 400 MU/min used in the primary maze measurements. Data acquisition and background correction were performed as described for the primary analysis. Figure 2 shows the Ir‐192 and LINAC measurement geometries.

### 2.3 Dead‐time measurement (two‐source method)

The effective dead time of the ABG counting chain was estimated using a first‐order two‐source approximation based on the non‐paralyzable model.^6^ Because the effective dead time evaluated here represents the behavior of the assembled counting chain, including the GM‐module output pulse and edge‐triggered counting at the Arduino external interrupt input, two Sr‐90 beta point sources that produced stable high count rates were used for this characterization.

The two sources had decay‐corrected activities of 2.92 and 2.50 kBq, calculated from their certified initial activities using an Sr‐90 half‐life of 28.8 years.^17^ Each source was positioned 1.5 cm from the GM tube using a fixed, reproducible geometry. Following the standard two‐source protocol, three count‐rate measurements were obtained: the first source alone (*m*_1_), both sources together (*m*_12_), and the second source alone (*m*_2_). The position of each source remained unchanged as the other was added or removed. A source‐free background measurement was also acquired but was not included in the dead‐time calculation because the background count rate (0.45 cps) was less than 0.4% of the single‐source count rates, and its inclusion changed the estimated dead time by less than the propagated standard uncertainty. Count rates were logged in 1‐s bins for at least 600 s per configuration, and the mean count rate over the stable acquisition interval was used for analysis.

Under the non‐paralyzable model, the recorded count rate, m, is related to the true count rate, n, by m = n/(1 + nτ), where τ denotes the effective dead time.

Under a first‐order approximation applicable when count losses are small, the effective dead time was estimated as follows:

$$\tau =\frac{m_{1}+m_{2}-\;m_{12}}{2m_{1}m_{2}}$$

Count‐rate standard uncertainties were estimated as $\sigma_{m}=\sqrt{m/t}$, assuming Poisson counting statistics over the 600 s acquisition time. The standard uncertainty of τ was propagated from $m_{1}$, $m_{2}$, and $m_{12}$ using the first‐order law of uncertainty propagation with the partial derivatives described by Domański et al.^18^ The resulting dead‐time estimate is reported in Section 3.4.

### 2.4 Energy‐response characterization

The intrinsic photon energy response of the ABG detector was characterized using three discrete‐energy gamma‐emitting radionuclides—Co‐57, Cs‐137, and Co‐60—spanning photon energies from approximately 122 keV to 1.33 MeV. Each sealed point source was positioned 2 cm from the GM tube using a fixed, reproducible geometry. A 5‐mm plastic filter was placed between the source and detector to pass the emitted gamma rays while reducing beta‐particle contributions; the measured response was therefore interpreted as gamma‐dominant. Source activities, decay‐corrected to the measurement date, were 922.65 kBq for Co‐57, 334.34 kBq for Cs‐137, and 57.36 kBq for Co‐60. Gamma‐ray energies and emission yields were obtained from standard nuclear decay data^17^: 122 and 136 keV for Co‐57 (combined yield, approximately 96%), 662 keV for Cs‐137 (85%), and 1173 and 1332 keV for Co‐60 (approximately 100% each).

For each source, count rates were logged in 1‐s bins for at least 600 s, and the mean count rate over the stable acquisition interval was used; one acquisition was performed per radionuclide. For each radionuclide, emission‐normalized detection efficiency was defined as the dead‐time‐corrected count rate divided by the total gamma‐photon emission rate, calculated from source activity and summed gamma‐emission yields assuming isotropic emission over 4π steradians.

To express detector response per unit dose, the response for each radionuclide was normalized to the corresponding ambient dose equivalent, H*(10), at the measurement geometry. Because source‐to‐detector distance and measurement geometry were identical for all three radionuclides, the common geometric factor cancelled in the relative comparison. The air‐kerma contribution of each emitted gamma line was calculated using the mass energy‐absorption coefficients for air^19^ and converted to H*(10) using photon air‐kerma‐to‐H*(10) conversion coefficients.^20^ For Co‐57 and Co‐60, H*(10) was calculated by summing the yield‐weighted contributions of the emitted gamma lines. Relative response per unit H*(10) was normalized to the Cs‐137 condition.

### 2.5 Data acquisition, background correction, and calibration‐coefficient estimation

ABG count‐rate outputs (CPM) and reference‐survey‐meter readings (µSv/h) were logged in real time to comma‐separated‐value files. Before each measurement session, both instruments were operated for 5 min under source‐free conditions, and background measurements were obtained in triplicate. The mean background value was subtracted from each measurement. Net signals were calculated as follows:

$$CPM_{net}=CPM_{raw}-CPM_{bg},\;{\dot{H}}_{net}^{SM}={\dot{H}}_{raw}^{SM}-{\dot{H}}_{bg}^{SM}$$

Observations with nonpositive values after background correction were excluded from the analysis.

The ABG‐estimated dose rate was calculated as follows:

$${\dot{H}}_{est}^{GM}=\frac{CPM_{net}}{CF}$$

where CF denotes the calibration coefficient in CPM per µSv/h. For each condition, the CF was estimated using a zero‐intercept regression model^5^, ^6^:

$${\dot{H}}_{net}^{SM}\approx b_{CF}\cdot CPM_{net}$$

Zero‐intercept regression was selected as the primary CF‐estimation method because both ABG CPM and reference‐survey‐meter dose rates were background‐corrected before calibration. Accordingly, the net ABG count rate was expected to approach zero as the net reference dose rate approached zero. To assess the potential effects of residual background fluctuation, system offset, and a nonzero baseline response, a free‐intercept regression was also performed as a sensitivity analysis for the CT 2‐m condition.

The regression‐based CF was then calculated as follows:

$$\;\hat{CF}=\frac{1}{{\hat{b}}_{CF}}$$

The 95% confidence intervals (CIs) for ${\hat{b}}_{CF}$ and $\hat{CF}$ were estimated using percentile bootstrap resampling with 1,000 resamples.^21^ For CT, the 2‐m coefficient was selected as the representative CF for low‐to‐moderate dose‐rate conditions. For Ir‐192 and the 6 MV LINAC maze, configuration‐ or distance‐specific CFs were estimated separately.

### 2.6 Agreement analysis and distance dependence

Agreement between ABG‐estimated and reference dose rates was evaluated using simple linear regression and Bland–Altman analysis.^22^, ^23^, ^24^ In the regression analyses, the reference‐survey‐meter reading was treated as the dependent variable and the ABG‐estimated dose rate as the independent variable. The slope, intercept, coefficient of determination (R^2^), and root‐mean‐square error (RMSE) were calculated.^7^

For Bland–Altman analysis, the between‐method difference (ABG system − reference survey meter) was plotted against the mean of the paired measurements, and the 95% limits of agreement (LoA) were calculated as the mean bias ± 1.96 standard deviations (SDs). The percentage error was defined as follows:

$$\%Error=100\times \frac{ABG-SM}{SM}$$

Distance dependence was additionally examined using log–log regression of dose rate against distance. Deviations from the ideal inverse‐square trend were interpreted in relation to scattering, shielding, and measurement geometry.

### 2.7 Portable deployment and survey

For field evaluation, a portable version of the system (ABG_P) was developed by integrating the Arduino‐based mainboard, GM tube, LCD, and battery power supply into a single enclosure (Figure S1).^6^, ^11^, ^13^, ^14^, ^15^ The device displayed real‐time CPM, the converted dose rate, and the measurement location or condition.

Following institutional review board approval, ABG_P units were deployed for 21 days in the radiation oncology, radiology, and nuclear medicine departments from December 2 to 23, 2025. Two devices were provided to each department for observational monitoring only and were not used for clinical decision‐making. Representative deployment sites are shown in Figure S2.^3^, ^4^

After the deployment period, staff who had used or directly encountered the device during routine work completed a self‐administered questionnaire. A total of 27 completed questionnaires were obtained. The questionnaire collected participant characteristics and included eight items rated on a five‐point Likert scale (S1–S8) and seven free‐text items (Q1–Q7). The quantitative items assessed usability, workflow compatibility, display clarity, usefulness relative to a conventional survey meter, perceived adequacy for detecting changes, perceived risk, contribution to radiation‐safety management, and intention for continued use. Item S6 was reverse‐coded. A composite acceptability and usefulness score was calculated as the mean of items S1–S5 and S7, whereas intention for continued use (S8) was designated the primary survey outcome.

### 2.8 Statistical analysis

#### 2.8.1 Detector‐validation analyses

For detector evaluation, ABG‐derived equivalent dose‐rate estimates were compared with reference‐survey‐meter measurements. For individual observations, an observation‐level count‐to‐dose ratio was calculated as background‐corrected ABG net CPM divided by the corresponding background‐corrected reference equivalent dose rate (CPM per µSv/h); this descriptive ratio is distinct from the scenario‐specific CF and was not used for dose‐rate conversion. ABG‐estimated equivalent dose rates were calculated by dividing net ABG CPM by the corresponding regression‐derived condition‐specific CF (Section 2.5), which was used for all CPM‐to‐dose‐rate conversions. Agreement between the ABG system and reference survey meter was assessed using linear regression, mean and percentage bias, and Bland–Altman analysis. Distance‐dependent variability in the LINAC maze was assessed by comparing distance‐specific CFs, mean biases, and LoA across measurement distances. Calibration uncertainty, measurement repeatability, and propagated uncertainty were also evaluated to support interpretation of the agreement analyses. Relative CF uncertainty was calculated as the half‐width of the 95% CI divided by the CF point estimate, that is, $\frac{CI_{\mathrm{upper}}-CI_{\mathrm{lower}}}{2\times 1.96\times CF}\times 100$. Measurement repeatability was assessed using the coefficient of variation (CV) of repeated net ABG CPM measurements within each measurement condition. The propagated relative uncertainty of the ABG‐estimated dose rate was calculated by combining relative CF uncertainty with the repeatability CV of net ABG CPM. Descriptive statistics are reported as the mean ± SD unless otherwise specified.

#### 2.8.2 Staff‐survey analyses

Participant characteristics were summarized as n (%), and questionnaire scores as the mean ± SD. The internal consistency of the composite acceptability and usefulness score was evaluated using Cronbach's alpha.^25^ Associations between questionnaire variables and the primary outcome, intention for continued use (S8), were assessed using Spearman rank correlation coefficients. Univariate linear regression analyses using standardized variables were performed to estimate standardized regression coefficients, 95% CIs, and p‐values for associations with S8. Free‐text responses were analyzed using codebook‐based thematic analysis, with multiple codes permitted for each response.^26^ Statistical analyses were performed using Python and IBM SPSS Statistics, version 29.0.9.0 (IBM Corp., Armonk, NY, USA). All statistical tests were two‐sided, with p < 0.05 indicating statistical significance.

### 2.9 Ethics

The staff‐survey component was approved by the local institutional review board (IRB No. 2026GR0016). All survey participants provided written informed consent.

## 3 RESULTS

### 3.1 CT scatter measurements at 120 kV

All CT scatter analyses used background‐corrected measurements (ABG system, 24 CPM; reference survey meter, 0.2 µSv/h). A common CF of 133 CPM per µSv/h was applied in the initial CT scatter comparison. This value was based on the zero‐intercept calibration estimate at 2 m (132.62 CPM per µSv/h, rounded to 133) rather than on the combined 1‐ and 2‐m data because the 2‐m condition showed greater stability and near‐zero mean bias. Mean bias was −13.21 µSv/h at 1 m and −0.66 µSv/h at 2 m, with lower error dispersion at 2 m (Table S1).

**FIGURE 3:**
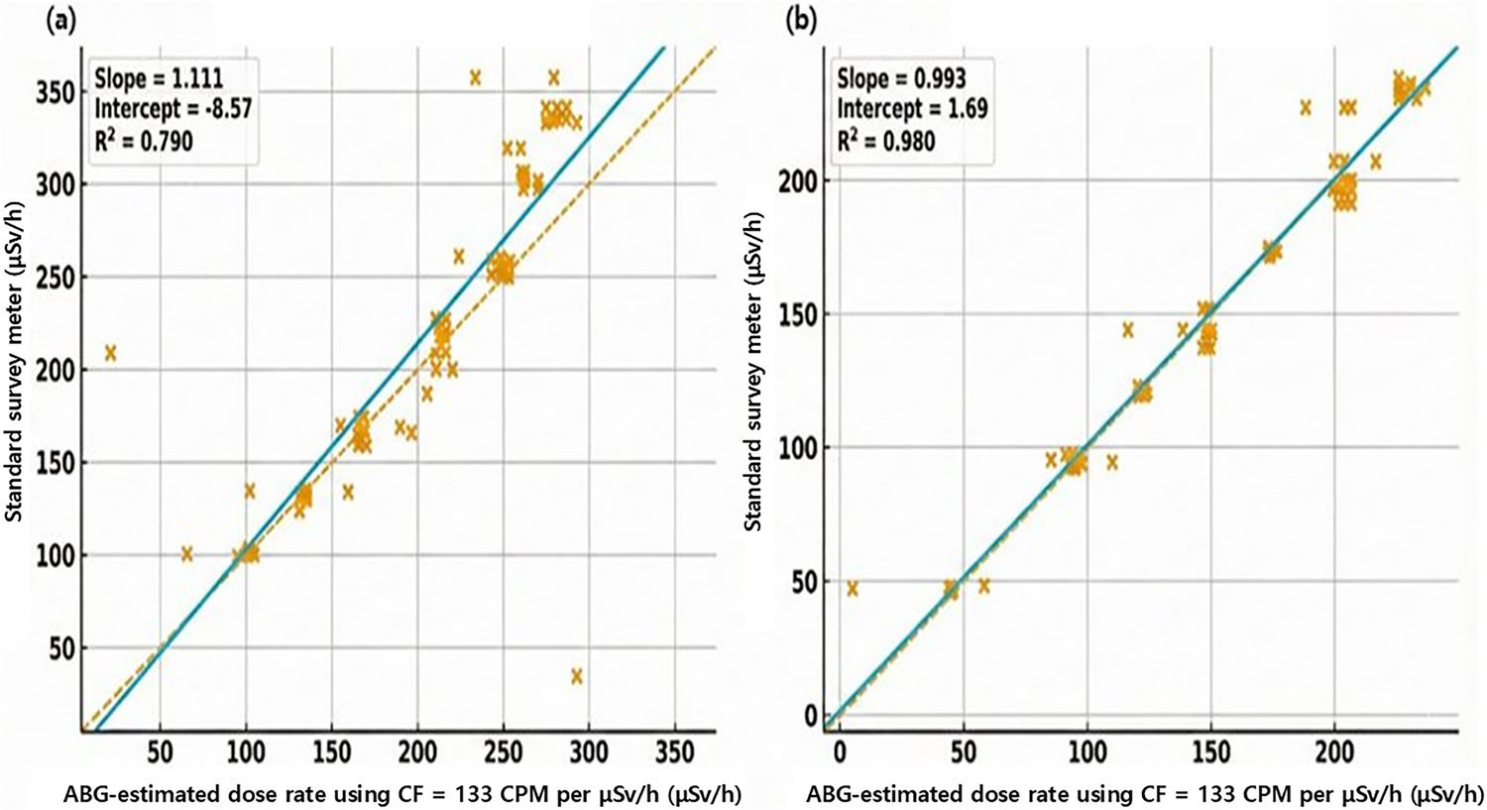
Comparison of ABG‐estimated and reference‐survey‐meter dose rates for CT scatter at 120 kV and 15–45 mA using a common CF of 133 CPM per µSv/h. Measurements were obtained at (a) 1 m and (b) 2 m from the CT gantry. Crosses indicate paired measurements, the dashed line represents the identity line (y = x), and the solid line represents the fitted regression line.

As shown in Figure 3, the ABG‐estimated values at 2 m more closely followed the identity line, whereas deviations were greater at 1 m, particularly at higher count rates.

**FIGURE 4:**
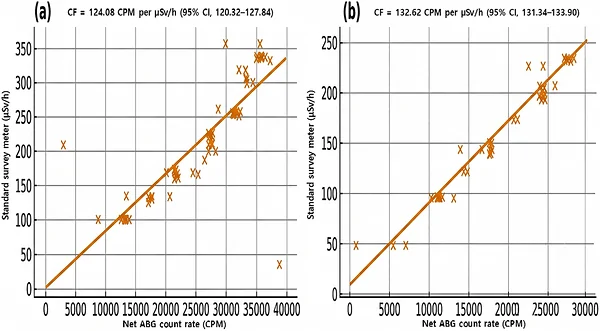
Zero‐intercept calibration for CT scatter at 120 kV and 15–45 mA. Calibration was based on the relationship between net ABG CPM and reference‐survey‐meter dose rate at (a) 1 m and (b) 2 m from the CT gantry. Crosses indicate paired measurements, and the solid line represents the fitted zero‐intercept regression.

Zero‐intercept regression yielded distance‐specific CF estimates of 124.08 CPM per µSv/h (95% CI, 120.32–127.84) at 1 m and 132.62 CPM per µSv/h (95% CI, 131.34–133.90) at 2 m (Figure 4). A free‐intercept regression model was also examined as a sensitivity analysis for the CT 2‐m condition. Allowing a nonzero intercept changed the estimated CF from 132.62 to 132.20 CPM per µSv/h, a difference of approximately −0.3%. The intercept was −0.55 µSv/h (*p* = 0.762), supporting approximate proportionality between background‐corrected net ABG CPM and reference dose rate under this measurement condition.

Bias became progressively more negative as tube current increased at 1 m but remained near zero across most tube‐current settings at 2 m (Figure S3). Bland–Altman analysis showed narrower LoA at 2 m and wider LoA at 1 m, indicating lower precision in the higher count‐rate condition (Figure S4).

### 3.2 Ir‐192 measurements

Two Ir‐192 configurations were evaluated: a shielded maze‐exit scatter‐dominant field and a sealed‐source near‐field condition. After configuration‐specific calibration, mean bias was −0.16 µSv/h in the shielded maze‐exit condition and 0.06 µSv/h in the sealed‐source near‐field condition, with narrow LoA in both geometries (Table S2).

**FIGURE 5:**
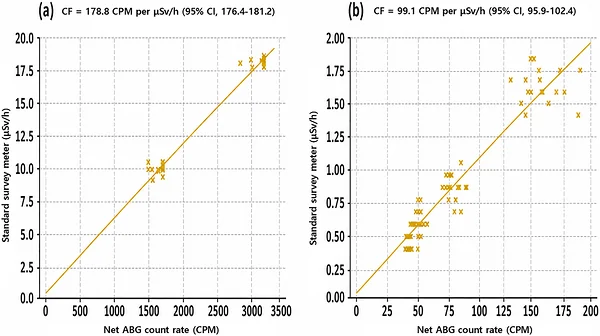
Zero‐intercept calibration of the ABG system for Ir‐192 measurements under two geometries: (a) shielded maze‐exit condition (*N* = 36; 100–200 cm) and (b) sealed‐source near‐field condition (*N* = 87; 25–150 cm). Crosses indicate paired measurements, and the solid line represents the fitted zero‐intercept regression.

Zero‐intercept regression yielded CFs of 178.8 CPM per µSv/h (95% CI, 176.4–181.2) for the shielded maze‐exit condition and 99.1 CPM per µSv/h (95% CI, 95.9–102.4) for the sealed‐source near‐field condition (Figure 5; Table S3).

**FIGURE 6:**
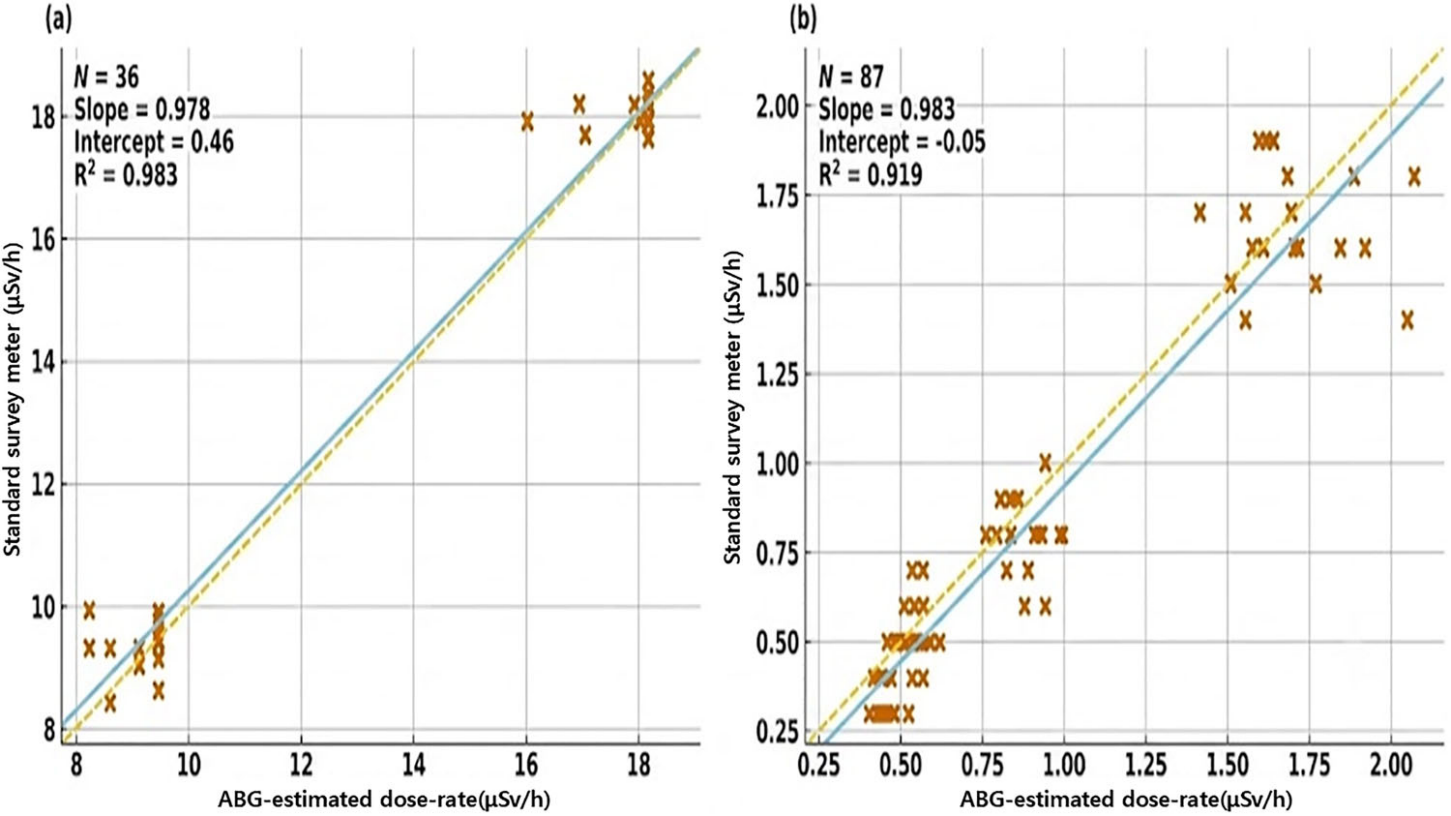
Comparison of ABG‐estimated and reference‐survey‐meter dose rates for Ir‐192 measurements using the configuration‐specific CFs shown in Figure 5. (a) Shielded maze‐exit configuration and (b) sealed‐source configuration. Crosses indicate paired measurements, the dashed line represents the identity line (y = x), and the solid line represents the fitted regression line.

As shown in Figure 6, the regression slopes approached unity in both configurations after application of the configuration‐specific CFs.

### 3.3 6 MV LINAC maze measurements

In the 6 MV LINAC maze, CFs varied by measurement distance despite identical beam settings. Zero‐intercept regression yielded CFs of 232.6, 211.0, and 145.2 CPM per µSv/h at 2, 4, and 6 m, respectively (Table S4).

**FIGURE 7:**
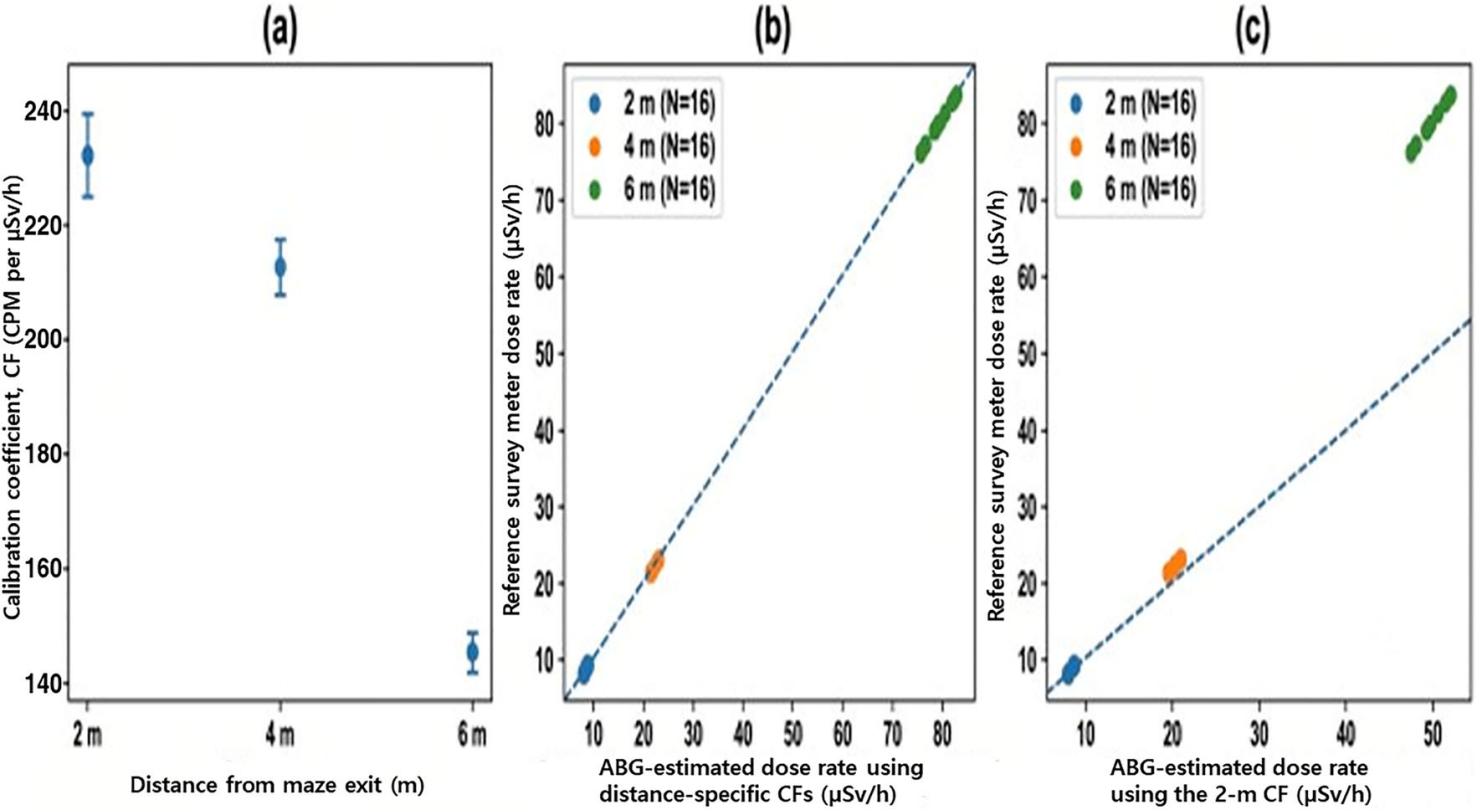
Distance dependence of the CF in the 6 MV LINAC maze and its effect on comparisons between ABG‐estimated and reference‐survey‐meter dose rates. (a) Distance‐specific CF estimates derived by zero‐intercept regression at 2, 4, and 6 m; points represent CF estimates, and error bars represent 95% CIs. (b) Comparison using distance‐specific CFs; colors denote measurement distance, and the dashed line represents the identity line. (c) Comparison using the CF derived at 2 m for all measurement distances.

As shown in Figure 7, applying distance‐specific CFs reduced bias relative to the reference survey meter across all distances. By contrast, applying the 2‐m CF at all distances produced greater bias at more distant points, indicating that a single distance‐invariant CF did not adequately represent the evaluated maze conditions.

With distance‐specific CFs, mean bias was 0.71 µSv/h at 2 m, −0.39 µSv/h at 4 m, and −0.15 µSv/h at 6 m. Although mean bias remained small at all distances, variability increased with distance, and the widest LoA occurred at 6 m (Table S5).

**FIGURE 8:**
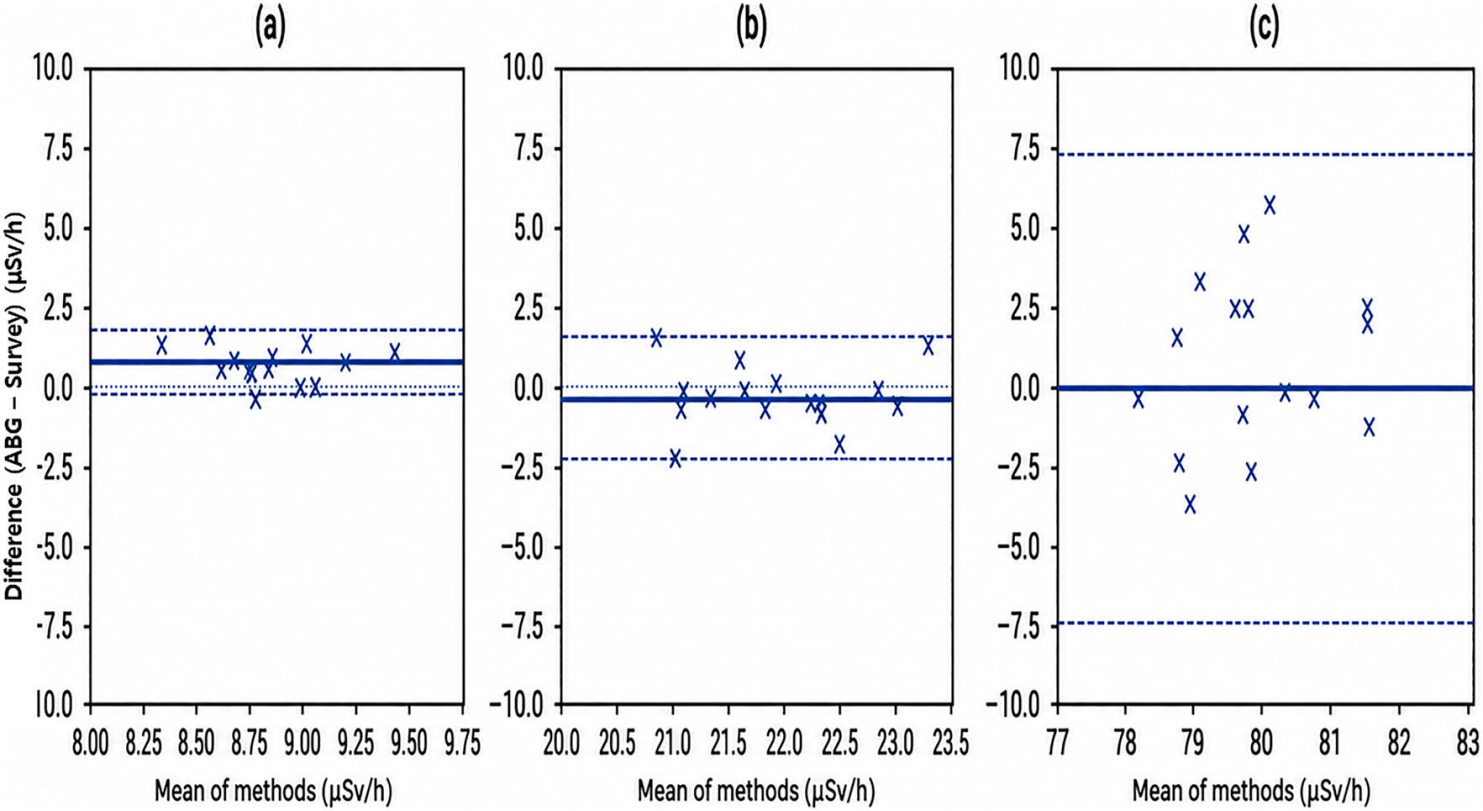
Bland–Altman plots comparing ABG‐estimated dose rates with reference energy‐compensated GM survey‐meter readings at (a) 2 m, (b) 4 m, and (c) 6 m in the 6 MV LINAC maze after application of distance‐specific CFs. Crosses indicate paired observations, the solid horizontal line represents mean bias, and the dashed horizontal lines represent the 95% LoA. Mean bias was near zero at all distances, whereas the LoA were widest at 6 m.

Bland–Altman plots showed near‐zero mean bias after distance‐specific calibration but wider dispersion at 6 m, indicating lower precision at greater maze distances (Figure 8).

An additional fixed‐location dose‐rate analysis was performed at the 2‐m maze position to evaluate CF stability across nominal dose rates. Table S6 summarizes the results. Across nominal dose rates of 100–600 MU/min, the CF ranged from 217.44 to 237.98 CPM per µSv/h, with a mean of 229.20 CPM per µSv/h and a CV of 3.04%. CF did not decrease monotonically as nominal dose rate increased, and marked count‐rate‐dependent response compression was not observed within the evaluated dose‐rate range at the 2‐m LINAC maze position.

### 3.4 Dead‐time measurement

Using the two‐source method described in Section 2.3, the effective dead time of the ABG counting chain was estimated at τ ≈ 258 µs, with a counting uncertainty of approximately 31 µs (12.1%, *k* = 1; Table S7). This first‐order two‐source estimate served as an empirical, model‐based indicator of count‐loss behavior under the evaluated measurement configuration and as the reference value for subsequent count‐loss estimates. At the count rates observed in the fixed‐location LINAC 2‐m dose‐rate analysis (≤47 counts/s), this dead time corresponded to an expected fractional count loss of less than approximately 1.5%, consistent with the limited CF variation across 100–600 MU/min.

### 3.5 Energy response

Emission‐normalized gamma detection efficiency, calculated as the dead‐time‐corrected count rate divided by the total gamma‐photon emission rate, increased across the radionuclide conditions: 8.98 × 10^−^^5^ counts per emitted photon for Co‐57, 2.47 × 10^−^^4^ for Cs‐137, and 4.51 × 10^−^^4^ for Co‐60. When expressed per unit dose, however, relative response was highest for the lowest‐energy radionuclide condition: compared with Cs‐137, response per unit ambient dose equivalent, H*(10), was approximately 1.8 for Co‐57 and 1.1 for Co‐60 (Table S8). These findings indicate greater per‐dose response to lower‐energy photons, consistent with the known energy dependence of uncompensated GM tubes and providing a plausible mechanistic explanation for the condition dependence of the CF.

### 3.6 Overall summary of condition‐specific calibration

Among the evaluated environments, CT scatter at 2 m showed the lowest bias and narrower error dispersion with the common CF, whereas the Ir‐192 and 6 MV LINAC maze measurements required configuration‐ or distance‐specific calibration. Table 2 summarizes the condition‐specific CFs and mean biases across all measurement scenarios.

**TABLE 2:** Condition‐specific calibration coefficients and mean bias across all measurement scenarios.

Condition/modality	Geometry/setup	Radiation field/configuration	N^e^	CF, CPM per µSv/h^a^ (95% CI)	Mean bias, µSv/h^b^, mean ± SD
CT (120 kV)	1 m; 15–45 mA	CT scatter, near gantry	122	124.1 (120.3–127.8)	−13.21 ± 35.18
CT (120 kV)	2 m; 15–45 mA	CT scatter, farther from gantry	122	132.6 (131.3–133.9)	−0.66 ± 8.42
Ir‐192	100–200 cm; shielded maze exit	Scatter‐dominant field at the maze exit	36	178.8 (176.4–181.2)	−0.16 ± 0.58
Ir‐192	25–150 cm; sealed source near the exit	Sealed‐source near field	87	99.1 (95.9–102.4)	0.06 ± 0.14
6 MV LINAC maze	2 m, near exit; 10 × 10 cm^2^ field	High‐energy maze field, near exit	16	232.6 (226.0–239.2)	0.71 ± 0.49
6 MV LINAC maze	4 m, mid‐maze; 10 × 10 cm^2^ field	High‐energy maze field, intermediate distance	16	211.0 (206.5–215.6)	−0.39 ± 0.96
6 MV LINAC maze	6 m, deep maze; 10 × 10 cm^2^ field	High‐energy maze field, deep maze	16	145.2 (141.8–148.5)	−0.15 ± 3.82

All values were calculated after background correction. The background values were 24 CPM for the ABG system and 0.2 µSv/h for the reference survey meter. The reference instrument was an energy‐compensated GM survey meter (Model 3005; Ludlum Measurements, USA). *N* = number of background‐corrected paired 1‐s observations pooled across settings and the three repetitions after exclusion of nonpositive net values (Section 2.5).

^a^ CFs were estimated using origin‐constrained regression; values in parentheses are 95% CIs.

^b^ Mean bias was calculated as the ABG‐estimated dose rate minus the reference‐survey‐meter dose rate; positive and negative values indicate overestimation and underestimation, respectively, by the ABG system.

Table S9 summarizes the uncertainty analysis. Relative CF uncertainty ranged from 0.49% to 1.66% across scenarios, whereas propagated uncertainty was influenced primarily by the repeatability of net ABG CPM. The Ir‐192 sealed‐source condition had the highest propagated uncertainty, corresponding to greater relative counting variability at low count rates.

### 3.7 Staff‐survey results

The survey analysis included 27 respondents, whose characteristics are summarized in Table S10. The mean composite acceptability and usefulness score was 4.01 (SD, 0.58; Cronbach's α, 0.913), and the mean score for continued use and dissemination intention (S8) was 4.59 (SD, 0.69; Table S11). Lower perceived risk and greater perceived stability showed the strongest association with S8; display clarity, perceived usefulness relative to a conventional survey meter, and perceived adequacy for trend detection were also positively associated with S8 (Table S11).

Free‐text responses indicated that respondents primarily viewed the portable system as a supplementary tool for controlled‐area patrols, CT scatter or leakage checks, and LINAC or maze screening rather than as a replacement for high‐precision reference instruments. Commonly reported implementation needs included greater reading stability, clearer standard operating procedures, defined cross‐checking criteria, site‐specific CF management, periodic verification, and training materials. Table S12 and Figure S5 provide the theme frequencies, operational definitions, and qualitative framework.

## 4 DISCUSSION

### 4.1 Principal findings

This study showed that the ABG system produced low mean bias relative to a calibrated energy‐compensated GM survey meter when condition‐specific CFs were applied, particularly for CT scatter at 2 m and both Ir‐192 configurations under low‐to‐moderate dose rates.^5^, ^6^, ^15^ In the 6 MV LINAC maze, mean bias was also low, but only after the CFs were stratified by distance.

These findings indicate that ABG‐system performance depended on the specific measurement scenario rather than on a single universal CF.^5^, ^6^, ^7^ Differences among CT, Ir‐192, and the 6 MV LINAC maze likely reflect variation in geometry, shielding, scatter contribution, and the detector's intrinsic energy dependence.^5^, ^6^, ^7^ Bench characterization using discrete‐energy gamma sources showed greater response per unit dose at lower photon energies (Section 3.5; Table S8), indicating that effective energy spectrum may contribute to the observed condition dependence of the CF alongside geometric and shielding effects. Thus, the principal practical implication is that a low‐cost GM‐based device may provide dose‐rate estimates with sufficiently low bias for supplementary monitoring when calibration is linked to the measurement conditions.^14^, ^15^ These findings support a condition‐specific calibration approach rather than a single global count‐to‐dose conversion constant.^5^, ^11^, ^15^

### 4.2 High‐count‐rate limitations and clinical implications

The principal technical limitation of the ABG system was systematic under‐response at high count rates, most clearly observed in the 1‐m CT measurements, where negative bias increased with tube current. This pattern is consistent with count loss related to GM dead time and pulse overlap and may also reflect the simpler signal‐processing architecture of the ABG system relative to the reference instrument.^6^, ^8^

As described in Section 2.3, the effective dead time (τ ≈ 258 µs) was estimated using the first‐order two‐source approximation under a non‐paralyzable dead‐time model, under which fractional count loss is expressed as (n − m)/n = mτ. Using this relationship, expected fractional count loss was estimated across the count‐rate ranges encountered in this study. At count rates of approximately 8–47 counts/s in the fixed‐location 2‐m LINAC analysis, expected count loss remained below approximately 1.5%, consistent with the limited CF variation across 100–600 MU/min (Table S6). By contrast, at 1 m from the CT gantry, where count rates reached several hundred counts per second, expected loss increased to approximately 5%–10% and rose with tube current. At the deepest maze position of 6 m, the recorded count rate was approximately 194 counts/s, corresponding to an expected count loss of approximately 5% under the same model (Table S5). This estimated loss was smaller than the approximately 38% reduction in CF between the 2‐ and 6‐m maze positions, suggesting that count‐rate loss contributed only partly to the observed distance dependence.

Because count loss increases progressively with count rate, a single condition‐specific CF obtained by zero‐intercept regression can absorb the mean loss within a scenario but cannot fully track the larger loss at the upper end of a wide, high‐rate dynamic range.^6^ Accordingly, the increasing negative bias with tube current at CT 1 m is consistent with response decrease at high count rates, whereas the lower and narrower count‐rate range at CT 2 m keeps the response closer to linear (Figure 3; Figure S3; Table S1). Similarly, the relatively low count‐rate range at the fixed 2 m LINAC maze position is consistent with the near‐constant CF observed across 100–600 MU/min (Table S6). This interpretation does not negate the distance dependence of the CF across the 2, 4, and 6 m maze positions, which likely reflects additional effects of measurement geometry, shielding, and radiation field composition (Figure 7; Tables S4,S5).^5^, ^6^, ^7^ The effective dead‐time estimate (Section 3.4) and non‐paralyzable model together served as an interpretive framework rather than a per‐reading correction, since the condition‐specific CFs already absorb the mean count loss within each measurement condition.

The same‐location LINAC dose‐rate analysis (Section 3.3) provided further empirical support for this interpretation, minimizing confounding effects related to detector position, shielding geometry, and energy spectrum; similarly, the free‐intercept sensitivity analysis for the 2‐m CT condition (Section 3.1) again showed little model dependence. These findings support approximate proportionality at lower count rates, in contrast to the 1‐m near‐gantry CT condition (Figure 4).

These findings also have practical implications for system use. Because the CT measurements were limited to 120 kV and tube currents of 15–45 mA, these data do not support a universal numerical threshold. Instead, near‐gantry CT measurements, increasing tube‐current settings, and readings approaching the upper evaluated count‐rate range should prompt cross‐verification with a calibrated survey meter. Based on the measured effective dead time of approximately 258 µs, ABG count rates of roughly 180–360 counts/s correspond to estimated count losses of approximately 5%–10%, whereas the fixed‐location analysis directly evaluated count rates only up to approximately 47 counts/s. Under such conditions, the ABG system should be used primarily for supplementary trend monitoring or screening rather than independent quantitative dose‐rate assessment.

Accordingly, the ABG system should be regarded as an auxiliary instrument for routine surveillance, education, and distributed monitoring rather than a replacement for regulatory‐grade survey meters.^3^, ^4^, ^9^, ^11^ When high count rates are suspected, quantitative use should include safeguards such as increasing the source‐to‐detector distance and cross‐verifying readings with a standard survey meter.^3^, ^5^, ^8^ Firmware‐based dead‐time correction may improve performance, but it requires device‐specific validation that was not performed in this study.^6^ The propagated uncertainty analysis was consistent with these interpretations. Higher propagated uncertainty was observed under conditions with greater count‐rate instability or more complex geometry, including the 1‐m CT, sealed‐source Ir‐192, and 6‐m LINAC maze measurements (Table S9). Thus, condition‐specific CFs reduced systematic bias but did not eliminate uncertainty arising from count‐rate repeatability, residual background variation, or detector‐response limitations.

The fixed 1‐s counting interval was selected to support real‐time monitoring and responsive display and logging rather than maximize statistical stability. Longer counting or averaging intervals could reduce random count fluctuations and improve temporal stability, particularly at low dose rates.^6^ However, longer averaging intervals would not eliminate count loss caused by GM dead time or pulse overlap at high count rates; they would primarily smooth temporal variability in the displayed dose‐rate estimate. Alternative counting intervals and moving‐average strategies were not systematically evaluated and warrant further assessment, particularly for low‐count‐rate stability and saturation‐prone conditions.

### 4.3 User acceptability and implementation considerations

Respondents viewed the ABG_P system as a supplementary monitoring tool, valuing immediate readings, portability, and action‐oriented screening, but also emphasized the need for clearer interpretation criteria, cross‐checking procedures, and device‐management protocols.

Taken together, these findings suggest a potential hybrid implementation model in which reference‐grade instruments are retained for formal verification and low‐cost ABG systems are used as distributed supplementary monitoring nodes.^4^, ^9^, ^10^, ^11^ Given the scalability and connectivity of Arduino‐based platforms, such a model may be particularly applicable in settings that require broader spatial coverage.^4^, ^10^, ^11^

### 4.4 Limitations

This study had several limitations. First, the evaluated radiation fields were limited to CT at 120 kV, Ir‐192, and a 6 MV LINAC maze using a standardized 10 × 10 cm^2^ field rather than a maximum‐scatter configuration; the reported CFs therefore should not be extrapolated to other beam qualities, radionuclides, device configurations, or worst‐case shielding surveys. The energy‐response characterization (Section 2.4) was similarly limited to three discrete‐energy points, one fixed geometry, and one acquisition per radionuclide without a repeat‐based uncertainty estimate, so it provides mechanistic support for condition‐dependent CFs rather than a quantitative predictor of their values across clinical photon spectra.

Second, only one reference survey meter was used, and its energy and temporal response characteristics may have contributed to residual uncertainty. Third, metrological performance was evaluated using a single GM module; inter‐device and inter‐lot variability were not assessed, since the portable deployment evaluated usability and workflow feasibility rather than agreement among multiple ABG units. The reported CFs should therefore be considered unit‐specific, and between‐device variation in GM‐tube sensitivity, plateau behavior, dead time, pulse processing, or high‐voltage performance could introduce systematic differences unless multi‐device deployment includes unit‐specific acceptance testing, CF verification, and periodic constancy checks.

Fourth, the fixed 1‐s counting interval supported real‐time monitoring, but alternative counting or averaging intervals may be preferable for evaluating saturation‐prone conditions. The 78 counts/s recorded at the 4‐m maze position corresponds to an estimated count loss of approximately 2% under the dead‐time model (Section 4.2), reinforcing that geometry and shielding, rather than count‐rate loss, were the predominant sources of the observed distance dependence. Finally, because a free‐intercept sensitivity analysis was performed for only one CT condition, zero‐ and free‐intercept models were not compared across all scenarios, so residual offset or nonproportional detector response cannot be fully excluded; further work should extend this comparison across broader dose‐rate ranges and radiation fields.

The survey had additional limitations. It was conducted at one institution with 27 respondents, and most quantitative domains were assessed using single items. Accordingly, the questionnaire findings represent preliminary feasibility evidence rather than generalized user acceptance. Further evaluation should include multiple institutions, broader radiation scenarios, additional GM‐tube types, longer deployment periods, objective use logs, and validated multi‐item survey constructs.

## 5 CONCLUSIONS

Within the evaluated clinical radiation environments, the ABG monitoring system produced dose‐rate estimates with low mean bias relative to the calibrated reference survey meter when condition‐specific CFs were applied, particularly under low‐to‐moderate dose‐rate conditions; high‐count‐rate CT measurements showed systematic under‐response, and deep‐maze measurements showed lower precision despite bias correction.

These findings support further evaluation of the ABG system as a supplementary tool for leakage and scatter surveillance, repeated point monitoring, and radiation‐safety support under comparable conditions, rather than as a replacement for a regulatory‐grade survey meter. A potential deployment framework could link predefined CFs to specific measurement scenarios and require cross‐verification with a standard survey meter when high count rates are expected.

Although site‐ and device‐specific calibration is required, the present workflow provides a structured basis for further evaluation of open‐source GM‐based systems in clinical radiation‐safety practice.

## AUTHOR CONTRIBUTIONS

Jungsam Choi designed and assembled the ABG system, conducted the validation experiments, organized the dataset, and drafted the manuscript. Chai Hong Rim developed the analysis plan, performed the calibration and agreement analyses, and prepared the figures and tables. Hakyoung Kim coordinated the on‐site measurements, contributed to data collection and verification, and revised the manuscript. Wonho Lee supervised the study, conducted additional experiments, finalized the manuscript, and managed the submission process.

## CONFLICT OF INTEREST STATEMENT

The authors have no relevant conflicts of interest to disclose.

## ETHICAL APPROVAL AND CONSENT TO PARTICIPATE

This prospective study included a survey of radiation workers and was approved by the Institutional Review Board of Korea University Guro Hospital (IRB No. 2026GR0016). The study protocol and all study materials, including participant recruitment documents and survey questionnaires containing informed consent information, were reviewed and approved by the IRB. Written informed consent was obtained from all participants before their participation in the study. No personally identifiable information was collected during data acquisition or analysis, and the study procedures were designed to avoid interference with the participants’ routine occupational duties.

## Supporting information




Supporting Information

Supporting Information

## Data Availability

The datasets generated and analyzed during this study are available from the corresponding author upon reasonable request.
