# Supplementary material for: Application of a low‐cost Geiger–Müller monitoring system for clinical radiation environments
Source: J Appl Clin Med Phys. 2026 Aug 13;27(8):e70744. doi: 10.1002/acm2.70744 (PMC13473700; doi:10.1002/acm2.70744)
Supplement: Supplementary file 2 [file ACM2-27-e70744-s002.docx]

**Supplementary Material**

**Supplementary Figure Legends**

**Supplementary Fig. S1**. Reconfigured portable Arduino-based Geiger–Müller monitoring system (ABG_P) for routine operation and departmental loan, integrating the LCD and battery power into a single enclosure. The display shows the selected location label, CPM, and converted dose rate (µSv/h)

**Supplementary Fig. S2**. Example installations of the portable ABG unit (ABG_P) in medical radiation environments: **(a)** inside an equipment control room; **(b)** at a patient reception desk with radiation warning signage; and **(c)** beneath a monitor in an external radiation-equipment control room

**Supplementary Fig. S3**. Bias (ABG − SM) versus tube current for CT scatter measurements at (a) 1 m and (b) 2 m.

**Supplementary Fig. S4**. Bland–Altman plots for CT scatter measurements using the ABG system-converted dose-rate and the reference energy-compensated GM survey meter at (a) 1 m and (b) 2 m.

**Supplementary Fig. S5**. Structured framework summarizing qualitative themes related to use context, perceived value, limitations, and scale-up conditions for the portable ABG system.

**Supplementary Tables**

**Supplementary Table S1**. Agreement between ABG system dose-rate estimates and reference energy-compensated GM survey meter readings for CT scatter at 120 kV using a common CF of 133.

| Distance (m) | N | Tube current range (mA) | CPM, mean ± SD | ABG system dose-rate (µSv/h), mean ± SD | Survey meter dose-rate (µSv/h), mean ± SD | Mean bias (ABG - SM, µSv/h) | Percentage error, mean ± SD | R² | RMSE (µSv/h) |
| --- | --- | --- | --- | --- | --- | --- | --- | --- | --- |
| Overall | 244 | 15–45 | 22,588 ± 9,035 | 169.84 ± 67.93 | 176.77 ± 78.80 | −6.94 | −3.9 ± 48.62 | 0.88 | 27.83 |
| 1 | 122 | 15–45 | 26,044 ± 8,768 | 195.82 ± 65.92 | 209.03 ± 82.40 | −13.21 | −6.3 ± 68.22 | 0.79 | 37.58 |
| 2 | 122 | 15–45 | 19,133 ± 7,936 | 143.86 ± 59.67 | 144.52 ± 59.85 | −0.66 | −0.92 ± 9.55 | 0.98 | 8.45 |

**Supplementary Table S2**. Summary of Ir-192 measurements and agreement statistics by configuration.

| Condition | N | Distance (cm) | CPM, mean ± SD | ABG-estimated dose rate (µSv/h), mean ± SD | Survey meter dose rate (µSv/h), mean ± SD | Mean bias (ABG - SM, µSv/h) ± SD | Bias SE | 95% LoA (µSv/h) |
| --- | --- | --- | --- | --- | --- | --- | --- | --- |
| Covered/Maze exit | 36 | 100–200 | 2442 ± 805 | 13.54 ± 4.46 | 13.70 ± 4.40 | −0.16 ± 0.58 | 0.097 | [−1.30, 0.99] |
| Sealed source near field | 87 | 25–150 | 81 ± 46 | 0.87 ± 0.49 | 0.81 ± 0.50 | 0.06 ± 0.14 | 0.015 | [−0.22, 0.34] |

SE, standard error. Bias was calculated as ABG **-** SM

**Supplementary Table S3**. Origin-constrained calibration results for Ir-192 by measurement configuration.

| Condition | N | Slope, b_CF (=1/CF) | 95% CI lower for b_CF | 95% CI upper for b_CF | Estimated CF | 95% CI lower for CF | 95% CI upper for CF |
| --- | --- | --- | --- | --- | --- | --- | --- |
| Covered/Maze exit | 36 | 0.005592 | 0.005517 | 0.005668 | 178.8127 | 176.3989 | 181.2265 |
| Sealed source near field | 87 | 0.010087 | 0.009758 | 0.010415 | 99.1421 | 95.9123 | 102.3719 |

Calibration was performed by using a zero-intercept model that related the survey meter dose rate to the net CPM from the ABG system. The calibration coefficient was calculated as CF = 1 / b_CF

**Supplementary Table S4**. Origin-constrained calibration results for the 6 MV linear accelerator maze by distance.

| Distance (m) | N | Slope, b_CF (= 1/CF) | Estimated CF | 95% CI lower for b_CF | 95% CI upper for b_CF | 95% CI lower for CF | 95% CI upper for CF |
| --- | --- | --- | --- | --- | --- | --- | --- |
| 2 | 16 | 0.004299 | 232.5883 | 0.004178 | 0.004421 | 226.0260 | 239.1507 |
| 4 | 16 | 0.004739 | 211.0370 | 0.004637 | 0.004840 | 206.5169 | 215.5571 |
| 6 | 16 | 0.006888 | 145.1840 | 0.006728 | 0.007047 | 141.8234 | 148.5445 |

**Supplementary Table S5**. Agreement between the ABG system-derived and reference energy-compensated GM survey meter dose rates in the 6 MV LINAC maze by distance.

| Distance (m) | N | CPM, mean ± SD | ABG dose rate (µSv/h), mean ± SD | Reference survey meter dose rate (µSv/h), mean ± SD | Mean bias (ABG - reference, µSv/h) ± SD | Bias SE | 95% LoA (µSv/h) |
| --- | --- | --- | --- | --- | --- | --- | --- |
| 2 | 16 | 1974 ± 71 | 9.20 ± 0.33 | 8.49 ± 0.41 | 0.71 ± 0.49 | 0.126 | [−0.24, 1.66] |
| 4 | 16 | 4681 ± 213 | 21.83 ± 0.99 | 22.22 ± 0.44 | −0.39 ± 0.96 | 0.239 | [−2.26, 1.48] |
| 6 | 16 | 11641 ± 513 | 80.18 ± 3.53 | 80.33 ± 1.45 | −0.15 ± 3.82 | 0.955 | [−7.63, 7.34] |
| Overall | 48 | 6186 ± 4126 | 37.07 ± 31.31 | 37.62 ± 31.53 | 0.06 ± 2.29 | 0.331 | [−4.44, 4.55] |

**Supplementary Table S6**. Same-location LINAC dose-rate analysis for CF stability at the 2 m maze position.

| Nominal dose rate (MU/min) | N | Survey meter reading (µSv/h), mean ± SD | ABG CPM, mean ± SD | Net survey meter reading (µSv/h), mean ± SD | ABG net CPM, mean ± SD | CF, CPM per µSv/h, mean ± SD |
| --- | --- | --- | --- | --- | --- | --- |
| 100 | 3 | 2.33 ± 0.07 | 487.88 ± 17.2 | 2.13 ± 0.07 | 463.88 ± 17.2 | 217.44 ± 6.8 |
| 200 | 3 | 3.90 ± 0.09 | 863.80 ± 22.5 | 3.70 ± 0.09 | 839.80 ± 22.5 | 226.97 ± 5.9 |
| 300 | 3 | 6.10 ± 0.12 | 1428.10 ± 29.0 | 5.90 ± 0.12 | 1404.10 ± 29.0 | 237.98 ± 5.3 |
| 400 | 3 | 9.07 ± 0.15 | 2074.93 ± 31.6 | 8.87 ± 0.15 | 2050.93 ± 31.6 | 231.31 ± 4.6 |
| 500 | 3 | 10.40 ± 0.17 | 2403.59 ± 36.0 | 10.20 ± 0.17 | 2379.59 ± 36.0 | 233.29 ± 4.3 |
| 600 | 3 | 12.57 ± 0.19 | 2845.96 ± 44.2 | 12.37 ± 0.19 | 2821.96 ± 44.2 | 228.19 ± 4.0 |

ᵃ CF, calibration coefficient; ABG, Arduino-based Geiger–Müller monitoring system.
ᵇ Measurements were performed at the LINAC maze 2 m position using 6 MV photons and a 10 × 10 cm² field.
ᶜ Net values were calculated after background correction.
ᵈ CF was calculated for each replicate as ABG net CPM divided by the corresponding net survey-meter dose rate; values are reported as mean ± SD.

**Supplementary Table S7.** Two-source estimate of the effective dead time of the ABG counting chain using the first-order non-paralyzable approximation, with counting-statistics uncertainty.

| Symbol | Configuration | Count rate *m* (cps) | Acquisition time *t* (s) | Standard uncertainty σ_m_ (cps) |
| --- | --- | --- | --- | --- |
| *m_1_* | Source 1 alone | 120 | 600 | 0.447 |
| *m_2_* | Source 2 alone | 113 | 600 | 0.434 |
| *m_12_* | Both sources together | 226 | 600 | 0.614 |

| Derived quantity | Value |
| --- | --- |
| Numerator *m_1_+m_2_-m_12_* | 7 cps |
| Denominator 2*m_1_m_2_* | 27,120 cps² |
| Effective dead time τ | 258 µs |
| Standard uncertainty *uτ* | 31 µs, 12.1%, *k*=1 |
| Approximate coverage interval *k*=2 | 196–321 µs |

Two Sr-90 beta point sources with decay-corrected activities of 2.92 and 2.50 kBq were used; each source was positioned 1.5 cm from the GM tube in a fixed, reproducible geometry.

Count rates were logged in 1 s bins for 600 s per configuration, and the mean count rate over the acquisition period was adopted. The effective dead time was estimated using the first-order two-source approximation for a non-paralyzable counting system, $\tau=(m_{1}+m_{2}-m_{12})/(2m_{1}m_{2})$.

Count-rate standard uncertainties were estimated as $\sigma_{m}=\sqrt{m/t}$, assuming Poisson counting statistics over the 600 s acquisition time. The standard uncertainty of $\tau$was propagated from $m_{1}$, $m_{2}$, and $m_{12}$using the first-order law of uncertainty propagation with the partial derivatives described by Domański et al.^18^

**Supplementary Table S8.** Energy-response characterization of the ABG detector (2 cm, 5 mm plastic filter; dead-time corrected)

| Nuclide | Activity (kBq)ᵃ | Gamma energy, keV (yield) | Emission-normalized efficiency (counts/photon)ᵇ | Relative response per H*(10)ᶜ |
| --- | --- | --- | --- | --- |
| Co-57 | 922.65 | 122, 136 (≈96%) | 8.98 × 10⁻⁵ | 1.77 |
| Cs-137 | 334.34 | 662 (85%) | 2.47 × 10⁻⁴ | 1.00 |
| Co-60 | 57.36 | 1173, 1332 (≈100% each) | 4.51 × 10⁻⁴ | 1.11 |

ᵃ Decay-corrected to the measurement date.
ᵇ Counts per emitted gamma photon (4π), after non-paralyzable dead-time correction (τ ≈ 258 µs).
ᶜ Normalized to Cs-137 (662 keV); per unit air kerma the values are 2.37, 1.00, and 1.06, respectively.

**Supplementary Table S9**. Calibration coefficient uncertainty, measurement repeatability, and propagated relative uncertainty by measurement scenario.

| Scenario | N | | CF, CPM per µSv/h | 95% CI for CF | Relative CF uncertainty (%) | Repeatability CV of ABG net CPM (%) | Propagated relative uncertainty of ABG-estimated dose rate (%) |
| --- | --- | --- | --- | --- | --- | --- | --- |
| CT 1 m | 122 | | 124.08 | 120.32–127.84 | 1.55 | 4.77 | 5.01 |
| CT 2 m | 122 | | 132.62 | 131.34–133.90 | 0.49 | 3.40 | 3.44 |
| Ir-192 covered/maze-exit | 36 | | 178.81 | 176.40–181.23 | 0.69 | 4.16 | 4.22 |
| Ir-192 sealed-source | | 87 | 99.14 | 95.91–102.37 | 1.66 | 12.65 | 12.76 |
| LINAC 2 m | | 16 | 232.59 | 226.03–239.15 | 1.44 | 0.81 | 1.65 |
| LINAC 4 m | | 16 | 211.04 | 206.52–215.56 | 1.09 | 1.23 | 1.64 |
| LINAC 6 m | | 16 | 145.18 | 141.82–148.54 | 1.18 | 2.08 | 2.39 |

^a^ CF, calibration coefficient; CV, coefficient of variation.

^b^ Relative CF uncertainty was estimated from the 95% CI of the CF as $(upper CI - lower CI)/(2\times1.96\times CF)\times100$.

^c^ Repeatability CV was calculated from repeated ABG net CPM measurements within the same measurement condition.

^d^ Propagated relative uncertainty of the ABG-estimated dose rate was calculated by combining the repeatability CV of ABG net CPM and the relative standard uncertainty of the CF, $u_{\mathrm{rel}}(\mathrm{CF})$, in quadrature, assuming independent contributions.

**Supplementary Table S10**. Participant characteristics of survey respondents.

| Variable | Category | n | % |
| --- | --- | --- | --- |
| Profession | Radiologic technologist | 15 | 55.6 |
|  | Physician | 4 | 14.8 |
|  | Nurse | 5 | 18.5 |
|  | Medical physicist | 1 | 3.7 |
|  | Other | 2 | 7.4 |
| Years of experience | 0–4 years | 8 | 29.6 |
|  | 5–10 years | 11 | 40.7 |
|  | 11–19 years | 6 | 22.2 |
|  | ≥20 years | 2 | 7.4 |
| Main work area | Radiation area | 3 | 11.1 |
|  | Controlled area | 23 | 85.2 |
|  | Other | 1 | 3.7 |
| Frequency of use of conventional portable radiation survey meters | Daily | 18 | 66.7 |
|  | 2–3 times per week | 7 | 25.9 |
|  | Weekly | 2 | 7.4 |

**Supplementary Table S11**. Domain-level descriptive statistics and associations with continued use/intention to disseminate (S8) among survey respondents.

| Domain | No. of items | Mean (SD) | Cronbach’s α | Spearman ρ with S8 | b (standardized for S8) | 95% CI for b | P value |
| --- | --- | --- | --- | --- | --- | --- | --- |
| Usability | 1 | 4.19 (0.62) | — | 0.11 | 0.20 | [−0.21, 0.60] | 0.3225 |
| Workflow fit | 1 | 4.11 (0.70) | — | 0.17 | 0.28 | [−0.12, 0.67] | 0.1585 |
| Display clarity | 1 | 4.11 (0.64) | — | 0.30 | 0.40 | [0.02, 0.78] | 0.0394 |
| Usefulness versus commercial survey meter | 1 | 3.78 (0.80) | — | 0.34 | 0.42 | [0.05, 0.79] | 0.0293 |
| Trend detection sufficiency | 1 | 3.96 (0.71) | — | 0.34 | 0.39 | [0.01, 0.77] | 0.0424 |
| Low risk / stability (S6 reverse-coded) | 1 | 4.15 (0.77) | — | 0.53 | 0.52 | [0.17, 0.87] | 0.0054 |
| Safety contribution | 1 | 3.89 (0.70) | — | 0.19 | 0.24 | [−0.16, 0.64] | 0.2266 |
| Composite acceptability/usefulness | 6 | 4.01 (0.58) | 0.913 | 0.25 | 0.26 | [-0.14, 0.62] | 0.20 |

ᵃ The outcome variable, continued use/intention to disseminate (S8), had a mean (SD) of 4.59 (0.69).

**Supplementary Table S12**. Summary of qualitative themes identified across open-ended survey questions (Q1–Q7).

| Section | Theme (code) | Operational definition (what counts) | n (%) |
| --- | --- | --- | --- |
| Use context (Q1) | Controlled-area patrol & boundary checks | Routine rounds, boundary/entry monitoring, signage points in controlled areas | 9 (33.3) |
| Use context (Q1) | CT scatter/leakage spot-checks | Use around CT console/rooms/corridors/waiting area for scatter/leakage checking | 6 (22.2) |
| Use context (Q1) | LINAC/maze screening | Use in/near LINAC room or maze for safety checks and screening | 4 (14.8) |
| Use context (Q1) | Cross-check with commercial meter | Parallel measurement or re-check using a reference/conventional portable radiation survey meter | 3 (11.1) |
| Perceived value (Q2/Q4) | Immediate availability (rapid checks) | “Grab-and-check” use, rapid repeated checks across locations | 8 (29.6) |
| Perceived value (Q2/Q4) | Portability for frequent rounds | Lightweight/portable enabling frequent rounds and ad hoc checks | 5 (18.5) |
| Perceived value (Q2/Q4) | Trend/change detection | Focus on increase/decrease or relative changes rather than absolute values | 4 (14.8) |
| Perceived value (Q2/Q4) | Workflow efficiency | Reduced burden/time for routine monitoring; easier screening | 4 (14.8) |
| Perceived value (Q2/Q4) | Action/decision support | Led to actions (access control, workflow adjustment, additional verification) | 12 (44.4) |
| Perceived value (Q2/Q4) | Screening/triage & prioritization | Screen multiple locations first, then prioritize suspected points for further verification | 4 (14.8) |
| Limitations (Q3) | Reading fluctuation / stabilization time | Perceived variability; need to extend measurement time for stable reading | 6 (22.2) |
| Limitations (Q3) | Need SOP / cross-check criteria | Requests for written rules: when to use alone vs. cross-check and what to do | 6 (22.2) |
| Limitations (Q3) | Interpretation/over-trust risk | Concern about misinterpreting as absolute dose rate; need caution statements | 3 (11.1) |
| Limitations (Q3) | Label/readability improvements | Requests to clarify labels, location/condition info, or on-screen guidance | 3 (11.1) |
| Limitations (Q3) | Durability/log/alarm requests | Requests related to device durability/case, alarms/threshold alerts, or logging/recording | 6 (22.2) |
| Scale-up conditions (Q5–Q7) | Governance for loan/return | Need owner, tracking/log, loan/return protocol to avoid loss/misuse | 7 (25.9) |
| Scale-up conditions (Q5–Q7) | Feature upgrades | Requests for alarm/logging/readability/battery/durability improvements | 7 (25.9) |
| Scale-up conditions (Q5–Q7) | Standardized labels & site-specific CF | Fixed labels and consistent site-specific CF management | 5 (18.5) |
| Scale-up conditions (Q5–Q7) | Periodic verification/QC | Regular cross-verification and QC cycle to maintain confidence | 4 (14.8) |
| Scale-up conditions (Q5–Q7) | Training package | Quick guide/checklist and onboarding for new staff/students | 4 (14.8) |
| Education/policy integration (Q6) | Education use (new staff/students) | Use in training scenarios, demonstrating concepts with real readings | 15 (55.6) |
| Education/policy integration (Q6) | Policy/SOP integration | Inclusion as a supplementary tool in radiation safety policy/SOP | 12 (44.4) |
| Education/policy integration (Q6) | Mitigation measures | Warning text + cross-check rules + standardized scripts to prevent misuse | 9 (33.3) |

ᵃ Multi-coding was allowed; therefore, percentages do not sum to 100%
ᵇ Percentages were calculated using the total number of respondents (N = 27) as the denominator
ᶜ Operational definitions indicate the criteria used to assign each response to a thematic code
ᵈ SOP, standard operating procedure; QC, quality control
